# Commentary: The Effect of Corticosteroid Injection in the Treatment of Greater Trochanter Pain Syndrome

**DOI:** 10.12968/ijap.2023.0056

**Published:** 2024-10-02

**Authors:** M McCarney, C Brennan, S Bunting, S Hill, J Hill

**Affiliations:** Blackpool Teaching Hospitals NHS Foundation Trust; University of Central Lancashire

## Abstract

Greater Trochanteric Pain Syndrome is a broad term employed to characterize lateral hip pain originating from the structures attached to the greater trochanter of the femur. The documented decrease in work participation, elevated levels of pain and dysfunction impeding physical activity, and diminished quality of life align with those observed in individuals with severe hip osteoarthritis. Effectively managing Greater Trochanteric Pain Syndrome can present considerable challenges. Generally, patients with Greater Trochanteric Pain Syndrome tend to respond favourably to conservative management. However, regarding the conservative approach of using corticosteroid injections there is still some debate regarding the specific estimation of effect. A recent systematic review by Wang et al. (2022) was undertaken to explore the effect of corticosteroid injection in the treatment of Greater Trochanteric Pain Syndrome. This commentary seeks to critically assess the methodologies employed in the review conducted by Wang et al. (2022) and provide a broader understanding of the findings in the context of the four pillars of advanced practice; clinical practice, leadership, education and research.

## Introduction

Greater Trochanteric Pain Syndrome (GTPS) is a broad term used to describe lateral hip pain stemming from the structures attached to the greater trochanter of the femur (Pumarejo Gomez and Childress 2023). It affects an estimated 1.8 out of 1000 patients annually and is more common in women than men (Lievense et al. 2005). The reported reduced work participation levels, high levels of pain and dysfunction affecting physical activity, and reduced quality of life are comparable with those of people with severe osteoarthritis of the hip (Fearon et al. 2014). Currently there is little documented data on the economic impact of GTPs published but given that it is comparable to hip osteoarthritis it is plausible they are somewhat similar (Fearon et al. 2017). A recent study of the economic burden of hip osteoarthritis in the Netherlands reported an average of 159 sick leave calendar days and €12,482 in costs (Hardenberg et al. 2022).

Effectively managing GTPS can pose significant challenges (Reid 2016). Typically, patients with GTPS will respond well with conservative management (Pianka et al. 2021), which typically include physiotherapy, non-steroidal anti-inflammatory drugs, and corticosteroid injections (Pianka et al. 2021; Reid 2016). Regarding the latter of corticosteroid injections, there is still some debate regarding a specific estimation of effect with previous systematic reviews in this area but without meta-analysis (BarrattBrookes and Newson 2017). As highlighted in the Chartered Society of Physiotherapy’s guidance for advanced practice physiotherapists, injection therapy stands as one of the advanced skills utilized to offer patient-centred and sustainable care that is efficient, cost-effective, and clinically impactful (Chartered Society of physiotherapy 2018). Due to this lack of specific certainty of effect a recent systematic review by Wang et al (2022) was undertaken to explore the effect of corticosteroid injection (CSI) in the treatment of GTPS.

## Aim of commentary

This commentary aims to critically appraise the methods used within the review by Wang et al (2022) and expand upon the findings in the context of the four pillars of advanced practice; clinical practice, leadership, education and research.

## Critical appraisal/ key methods of Wang et al (2022)

Evaluating this systematic review using the MeaSurement Tool to Assess systematic Review (Amstar 2) indicated the fulfilment of 12 out of the 16 criteria (see Table 1 for critical appraisal and corresponding methods). The three main areas of concern were firstly the lack of justification of only including randomised controlled trials. Whereas this isn’t a substantial methodological issue, it is recommended in the Amstar tool to consider the pros and cons of including both randomised and non-randomised studies when they are available (Shea et al. 2017). Secondly the review did not indicate justifications for excluded studies. This lack of transparency makes it difficult to verify the reasons for exclusion. Thirdly, an evaluation of the consequences of potential bias on the provided estimates was absent. This absence makes it challenging to discern which specific elements of bias or an overall summary of bias risk might have influenced the outcomes. Lastly, there was a lack of disclosure regarding the funding sources for the studies that were incorporated. This data may offer insights into potential bias introduced by funding (Yaphe et al. 2001). Based upon this assessment it is deemed that this systematic review provides a comprehensive overview regarding the question of interest.

## Results of Wang et al. (2022)

A total of 80 records were identified of which after screening 8 RCTs were identified and subsequently included in a meta-analysis. Of these eight studies the main areas of concern of risk of bias were regarding the lack of blinding of participants (n=5), small sample bias (n=4), blinding of outcome assessor (n=3) and lack of allocation concealment (n=3).

When including all RCTs which assessed the effects of CSI compared to wait and see, usual care, or sham intervention there was no evidence of effect for short-term pain relief [1 to ≤ 6 weeks]. However, using a take one away analysis of a single RCT which was deemed to be a potential outlier and had a small sample size, there was a clinically and statistically significant large reduction in short-term pain (SMD − 0.78, 95%CI: − 1.04 to − 0.53); I^2^ = 0%). For the medium term [6 to ≤ 12 weeks] there was also a clinically and statistically significant moderate reduction of pain when compared to ‘wait and see’ and usual care for both analyses performed (SMD − 0.47, 95% CI: − 0.72 to −0.22, I^2^ = 0%). Irrespective of which tools were used from each study, the effect levels did notably change. For pain relief at six months there was no evidence of effect. However, at 12 months there was a borderline statistically significant but non-clinically statistically reduction in pain (SMD −0.27, 95% CI: − 0.52 to − 0.02, I^2^ = 0%). Similarly, there was a borderline statistically significant improvement in functionality at 12 months (SMD − 0.26, 95%CI: − 0.51 to − 0.02, I^2^ = 0%).

When comparing CSI to exercise there was no evidence of effect for short [1 to ≤ 6 weeks] and long-term [3-month and 6-month] pain relief. There was also no evidence of effect in improvement in functionality when comparing CSI to platelet-rich plasma in the short-term and medium-term. However, one RCT demonstrated a statistically significant reduction in improvement in long-term functionality (MD − 38.25, 95%CI: − 44.56 to − 31.94).

There was one RCT which found no evidence of difference between CSI versus dry needling in the short term. Additionally, there was one RCT which found a statistically significant reduction in short-term [1 to ≤ 6 weeks] (MD − 3.4, 95%CI: − 4.34 to − 2.46), moderate [4 months] (MD 1.3, 95%CI: 0.44 to 2.16) and long-term pain relief [15 months] (MD 2.9, 95%CI: 1.87, to 3.93).

## Commentary

### Clinical practice

The findings from this systematic review highlight that the current evidence base for GTPS management is sparse, although, these findings are broadly in keeping with other published literature and consensus opinion which recognizes the value of CSI in providing short and medium-term pain relief for GTPS (BarrattBrookes and Newson 2017; Reid 2016). Furthermore, the results from this systematic review (Wang et al. 2022) concerning the enduring impact of CSI on pain aligns with prior reviews, indicating that long-term effects may be small (BarrattBrookes and Newson 2017). However, due to the limited availability of evidence, these estimates carry a degree of uncertainty. Moreover, there was substantial heterogeneity observed concerning the short-term impact on pain. Regrettably, the limited number of studies prevented a thorough exploration of this heterogeneity. Nevertheless, upon examination of the three studies encompassed in the corticosteroid injection (CSI) versus ‘wait and see’ and usual care meta-analysis (short and medium term), they exhibited similar protocols and dosages. These included the administration of either 1 ml Betamethasone (5.7 mg/ml) or 1 ml Triamcinolone Acetonide (40 mg/ml), in combination with 1% or 2% Lidocaine, or 2 ml Bupivacaine, or 1 ml Marcaine. These doses are similar to what is commonly recommended for treating GTPS using CSI (Le and Sha 2023). Unfortunately, the systematic review by Wang et al (2022) did not assess adverse events of CSI as the review focused on pain and function. Previous reviews in this area have proposed that serious adverse events for CSI are relatively rare (Coombes, Bisset and Vicenzino 2010). However, this was based upon a relatively small evidence base and mainly focused on a single injection. When evaluating potential adverse events and risks, it is important for advanced practitioners to consider various factors, including the use of oral steroids, the frequency of injections, the specific types of steroids administered, and the timing of any significant surgical procedures (Stout, Friedly and Standaert 2019). Also, when addressing potential risks, it is important for advanced practitioners to engage in discussions with patients about potential outcomes such as pain, bleeding, infection, allergic reactions, and injury (Le and Sha 2023). In light of this uncertainty, and the diminishing effects observed over time and possible minor adverse events it is imperative to conduct consistent, regular and standardised monitoring of pain (Dydyk and Grandhe 2023). This practice is important to monitor the appropriate trajectory of pain for effective management.

### Management

Current NICE guidelines advocate CSI as a second line treatment to be considered for advanced practitioners if initial conservative treatments (reassurance, avoidance of provocative movements, ice, analgesia and lifestyle advice) do not provide adequate pain relief (National Institute for health and care excellence 2021). It is proposed that whenever feasible, CSI should be complemented by physiotherapy (National Institute for health and care excellence 2021). As highlighted in a previous systematic review on the management of GTPS, CSI may provide a pain relief window which may contribute to treatment outcomes (Reid 2016). Understanding the most effective moment to offer injection therapy for GTPS to maximize patient outcomes is paramount (McEvoy et al. 2013) for advanced practitioners. This would suggest that from a patient pathway perspective CSI may be needed initially at the start of a physiotherapy regimen. As an advanced practitioner, it is crucial to consider the debate regarding pre- or post-CSI when reflecting on managing this condition for each patient. The physiotherapy regimen would typically involve exercises that emphasize hip abduction, aimed at strengthening and stretching the gluteus medius and minimus muscles, or exercises for quadriceps strengthening, and iliotibial band stretching (National Institute for health and care excellence 2021; Speers and Bhogal 2017). It has been suggested that exercise regimens should be personalized to meet the specific needs of the individual, with an initial emphasis on enhancing gluteal strength and control (Christopher and Gurjit 2017). As hip control improves, the focus can then shift to strengthening the hip abductor muscles (Christopher and Gurjit 2017).

Despite these recommendations, there remains a significant degree of uncertainty regarding the most effective exercises for treating GTPS (Reid 2016). Further research in this field is needed to identify key moderating factors for this type of intervention (Reid 2016). Regrettably, the systematic review under consideration did not investigate the effectiveness of combining both CSI and exercise as a treatment approach. Nonetheless, when a direct comparison was made, no significant differences were observed in terms of pain improvement between CSI and exercise, both in the short-term and long-term. It is worth noting that these estimates had considerably wider confidence intervals, indicating a higher degree of uncertainty (Wang et al. 2022). Therefore, without additional studies, these results remain inconclusive. In a similar vein, when compared to platelet-rich plasma treatment, there was no evidence of a difference in the short-term and mid-term effects of pain reduction (Wang et al. 2022). Just like in the exercise comparison, these findings remain uncertain, primarily because of limited numbers of studies resulting in broad confidence intervals seen in these estimates. Given this uncertainty in comparing the two interventions, the decision-making process will heavily rely on patient preference and clinical experience (Szajewska 2018).

### Education

Within the review education was not a component of the conservative treatments compared or its importance explored. Contemporary evidence suggests that optimal treatment for tendinopathies requires a programme that targets the underlying pathology of tendinopathy using education for load management and exercise (Cook and Purdam 2012). Education helps to re-educate possible maladaptive beliefs and cognitions influencing pain and disability (Brodal 2017; Fenton Shih and Zolton 2015). The prevailing trend in current literature suggests that education is primarily implemented through in-person, face-to-face discussions (Hasani et al. 2021; JayaseelanWeber and Jonely 2019; Sancho et al. 2019). This approach fosters a dialogue centred on posing questions and providing answers to uncover implicit assumptions, thereby cultivating critical thinking skills (Wijma et al. 2016). This thorough evaluation is essential for pinpointing the patient’s requirements and customizing the educational material and presentation format accordingly (Wijma et al. 2016).

The inclusion of educational sessions at the beginning of treatment (e.g., through long sessions (Chimenti et al. 2023; JayaseelanWeber and Jonely 2019; Sancho et al. 2019) may be employed to promptly establish satisfactory adherence and a knowledge foundation that motivates physical activity and exercise. Incorporating an additional active educational learning strategy, where patients acquire knowledge through materials like videos and brochures, empowers therapists to customize interventions on an individual basis (Escriche-Escuder et al. 2023). This allows for a more focused exploration of aspects that patients find uncertain during subsequent face-to-face sessions (Escriche-Escuder et al. 2023). Regardless of the approach chosen, the focal point of education should centre on addressing the individual’s specific functional challenges. This involves diminishing the persistence of pain by minimizing provocation, especially during sustained or repetitive positions (Mellor et al. 2022). Additionally, enhancing pain self-efficacy can be achieved by improving comprehension of pathophysiology, understanding the effects of appropriate loading, alleviating fear, and offering clear, specific instructions (Mellor et al. 2022). To adopt these educational strategies, it is important for advanced practitioner to reflect upon their current pedagogical knowledge of these principles (Glaze 2001; NHS 2017). Where learning needs/development is identified, is important to seek out further pedagogical training within these domains (NHS 2017).

### Research

As an advanced practitioner, it is essential to employ robust methods when engaging in research to assess clinical practice (NHS 2017). The findings of this review highlight key methodological lessons that should be incorporated into future research in this area. Due to the limited number of studies and wide confidence intervals in the estimates presented for reduction of pain in the short, mid- and long-term, future research is required in assessing this effect. Because the evaluation of pain is subjective (Robinson et al. 1997), it is crucial for future research to strive, whenever feasible, for the blinding of both patients and assessors, as within the majority of studies included in this review this was unable to be undertaken. Additionally, a considerable proportion of the studies encompassed in this review were relatively small (Lin 2018). It is advisable for future research to endeavour, whenever feasible, to conduct larger-scale sampling across multiple centres (Das 2022). However, when undertaking a multicentre trial it important to try to standardised, as much as possible, the exact intervention procedure (Das 2022). Using such standardised approaches as highlighted by American College of Emergency Physicians may help to provide more repeatable and standardised interventions (American College of Emergency Physicians 2023). Furthermore, as highlighted in the review there was also notable variation in the comparator used within the included studies. Therefore, future research should try to use standardised placebo comparisons. It is important to note that there is some debate regarding what type of placebo is appropriate for injection therapy and this should be considered and explored prior to standardisation being recommended (Bar-OrRael and Brody 2017).

## CPD reflective questions

1- Would you carry out a Corticosteroid injection earlier in the pathway of care for patients who present with GTPS based on the review’s findings?
2- Would physiotherapy for GTPS provide better outcomes if the patients had a CSI prior to beginning rehabilitation?
3- How do we use education and to what degree do physiotherapists use education to enhance outcomes?
4- What further research would you carry out to advance the current evidence base for the management of GTPS?

Key Points

1- CSI can improve patients pain experience from GTPS in the short- and medium-term.
2- CSI offered earlier in the patient’s pathway of care for GTPS may enhance outcomes from physiotherapy rehabilitation.
3- Significant uncertainty remains regarding the most effective exercises to employ whilst rehabilitating patient’s with GTPS.

## Figures and Tables

**Table 1:** Critical appraisal of the review by Wang et al. (2022).

AMSTAR-2 items	Criteria/Methods
**1. Did the research questions and inclusion criteria for the review include the components of PICO?**	Yes − Only randomized controlled trials (RCTs) that enrolled adult participants with GTPS were included. Corticosteroid injection had to be one of the intervention groups in these trials, while the other interventions could be any conservative treatment for GTPS. Studies that included patients who had hip surgery, infection, acute trauma or Rheumatoid Arthritis were excluded. Screening, data extraction and assessment of bias was carried out by two reviewers independently.
**2. Did the report of the review contain an explicit statement that the review methods were established prior to the conduct of the review and did the report justify any significant deviations from the protocol?**	Yes − This systematic review was registered on PROSPERO prior to commencement and no deviations from the original post were undertaken.
**3. Did the review authors explain their selection of the study designs for inclusion in the review?**	No − There was no justification why only random controlled trials were included.
**4. Did the review authors use a comprehensive literature search strategy?**	Yes partially − A multi-database search was undertaken using MEDLINE, Embase and the Cochrane library from date of inception until 30th April 2021. However, there was no searching of trial registries.
**5. Did the review authors perform the study selection in duplicate?**	Yes − Two reviewers carried out study selection independently.
**6. Did the review authors perform data extraction in duplicate?**	Yes − Data extraction was carried out by two reviewers independently
**7. Did the review authors provide a list of excluded studies and justify the exclusions?**	No − A comprehensive list of excluded studies was not provided.
**8. Did the review authors describe the included studies in adequate details?**	Yes − All key variables were given regarding studies which were included in the review.
**9. Did the review authors use a satisfactory technique for assessing the risk of bias in the individual studies that were included in the review?**	Yes − Visual inspection of funnel plot was proposed, however a middle threshold of 10 studies were set which was not achieved.
**10. Did the review authors report on the sources of funding for the studies included in the review?**	No − The systematic review did not indicate the funding sources of included studies.
**11. If meta-analysis was performed did the review authors use appropriate methods for statistical combination of results?**	Yes − This meta-analysis employed both ‘fixed’ and ‘random’ methods of synthesis. The data was analysed utilising RevMan 5.4.1 software. Chi-Square test (Q test) and I2 were used to evaluate the statistical heterogenicity of the pooled data. An I2 value of >50% indicated that a random–effects model was employed, random. <%50 heterogenicity called for the use of a fixed-effect model being adopted.
**12. If meta-analysis was performed did the review authors assess the potential impact of RoB in individual studies on the results of the meta-analysis or other evidence synthesis?**	Yes − The systematic review proposed to do this, but unfortunately, they had less than 10 studies.
**13. Did the review authors account for RoB in individual studies when interpreting/discussing the results of the review?**	No − The risk of bias was assessed however it was not used in any type of subgroup analysis or sensitivity analysis to identify its effect regards to the estimates presented.
**14. Did the review authors provide a satisfactory explanation for, and discussion of any heterogeneity observed in the results of the review?**	Yes − Within the discussion they acknowledge that due to the potential risk of bias there is notable uncertainty in the estimates presented.
**15. If they performed quantitative synthesis did the review authors carry out an adequate investigation of publication bias (small study bias) and discuss its likely impact on the results of the review?**	Yes − They were unable to carry out a funnel plot assessment, there being less than 10 studies. However, they did carry out a sensitivity analysis of the take one away of the small study, which had a notable effect of the review’s findings.
**16. Did the review authors report any potential sources of conflict of interest, including any funding they received for conducting the review?**	Yes − There is a clear indication of funding for the systematic review.

## Key Points

1. Compared to usual care corticosteroid injection may have a large effect in reducing pain in the short-term [1 to ≤ 6 weeks] for adults with greater trochanter pain syndrome.
2. Over time, the pain-relieving benefits of corticosteroid injection diminishes.
3. There was no evidence of effect when comparing corticosteroid injection to exercise in both the short [1 to ≤ 6 weeks] and long term [3-month and 6- month] for adults with greater trochanter pain syndrome.
